# Rapid and simple SNP genotyping for *Bordetella pertussis* epidemic strain MT27 based on a multiplexed single-base extension assay

**DOI:** 10.1038/s41598-021-84409-0

**Published:** 2021-03-01

**Authors:** Kazunari Kamachi, Shu-Man Yao, Chuen-Sheue Chiang, Kentaro Koide, Nao Otsuka, Keigo Shibayama

**Affiliations:** 1 Department of Bacteriology II, National Institute of Infectious Diseases, Tokyo, Japan; 2 Center for Diagnostics and Vaccine Development, Centers for Disease Control, Taipei, Taiwan

**Keywords:** Clinical microbiology, Bacterial techniques and applications, Infectious-disease epidemiology

## Abstract

Multilocus variable-number tandem repeat analysis (MLVA) is widely used for genotyping of *Bordetella pertussis*, the causative bacteria for pertussis. However, MLVA genotyping is losing its discriminate power because prevalence of the epidemic MT27 strain (MLVA-27) is increasing worldwide. To address this, we developed a single nucleotide polymorphism (SNP) genotyping method for MT27 based on multiplexed single-base extension (SBE) assay. A total of 237 MT27 isolates collected in Japan during 1999–2018 were genotyped and classified into ten SNP genotypes (SG1 to SG10) with a Simpson’s diversity index (DI) of 0.79 (95% CI 0.76–0.82). Temporal trends showed a marked increase in the genotypic diversity in the 2010s: Simpson’s DI was zero in 1999–2004, 0.16 in 2005–2009, 0.83 in 2010–2014, and 0.76 in 2015–2018. This indicates that the SNP genotyping is applicable to the recently circulating MT27 strain. Additionally, almost all outbreak-associated MT27 isolates were classified into the same SNP genotypes for each outbreak. Multiplexed SBE assay allows for rapid and simple genotyping, indicating that the SNP genotyping can potentially be a useful tool for subtyping the *B. pertussis* MT27 strain in routine surveillance and outbreak investigations.

## Introduction

*Bordetella pertussis*, a highly transmissible gram-negative coccobacillus, is the etiologic agent of pertussis (whooping cough), a severe acute respiratory illness among children and a persistence cough among adolescents and adults. Vaccination is the most effective method for preventing and controlling pertussis; however, pertussis vaccines are unable to provide lifelong immunity^1–3^. The waning of vaccine-acquired immunity is thus a major contributor to pertussis resurgence. In most industrialized countries, the incidence of pertussis has increased despite high vaccination coverage, and the number of pertussis epidemics have also occurred^4–8^. In Japan, the number of reported pertussis cases of adolescents and adults significantly increased in the 2000s, and a nationwide epidemic of pertussis occurred during 2008–2010. Molecular strain typing revealed that certain strains of *B. pertussis* (genotype, MT27d) were associated with the pertussis epidemic^4^.

Molecular strain typing of infectious disease agents has become an important tool for their epidemiological surveillance and outbreak investigations. Strain typing methods currently used for *B. pertussis* are pulsed-field gel electrophoresis (PFGE)^9,10^, multilocus sequence typing (MLST)^11,12^, multilocus variable-number tandem repeat analysis (MLVA)^4,13,14^, and single nucleotide polymorphism (SNP) typing^15–20^. While PFGE has greater discriminatory power, it has the disadvantage in that the inter-laboratory comparison of PFGE patterns is difficult. MLST targets virulence-associated allelic genes of *B. pertussis* (commonly, *ptxP*, *ptxA1*, *prn*, and *fim3* alleles), whereas MLVA targets six variable number tandem repeat loci of *B. pertussis*. The MLST and MLVA methods allow for direct comparisons of *B. pertussis* isolates in different laboratories, with the MLVA method being the most widely used to study molecular epidemiology of this organism. However, MLST and MLVA are losing their discriminate power, since the MLVA type 27 (MT27) strain carrying the allele profile *ptxP3*/*ptxA1*/*prn2*/*fim3A* has become the predominant strain in *B. pertussis* population worldwide^4,10,21–24^. Currently, SNP genotyping based on whole-genome sequencing (WGS) is the most accurate method, and there are several reports of *B. pertussis* populations analyzed using whole-genome SNP analysis^15–18,21,25,26^. Recently, a novel WGS-based genotyping, called core genome MLST, was developed for global comparisons of *B. pertussis* isolates^27^. However, WGS has the disadvantage in that it is laborious, costly, and time-consuming for use in routine surveillance, especially in outbreak investigations.

Previously, a simple SNP genotyping method with 38 SNP targets was developed for *B. pertussis* using a single-base extension (SBE) assay^28^. The SBE assay is based on the incorporation of fluorescently labeled dideoxynucleotides (ddNTPs) into the 3′ end of allele-specific extension primers with a distinct length, and subsequent analysis with a capillary DNA sequencer. This assay allows for rapid and high-throughput genotyping. However, the SBE-based SNP genotyping had low discriminatory power for *B. pertussis* epidemic strain MT27, since the 38 SNP targets were selected based on the WGS data of various *B. pertussis* strains^28^. This limitation identifies the need for selecting polymorphic SNP markers among MT27 isolates in order to improve the discriminatory power for the MT27 strain.

In the present study, in order to easily subtype *B. pertussis* epidemic MT27 strain, we screened for polymorphic SNPs among MT27 isolates using their genome data and developed a novel SNP genotyping with 20 SNP targets based on multiplexed SBE assay. This genotyping system was evaluated with 237 *B. pertussis* MT27 isolates, and its applicability to outbreak investigations was assessed with outbreak-associated MT27 isolates.

## Methods

### Isolates and DNA preparation

We studied 237 *B. pertussis* MT27 isolates collected in Japan during 1999–2018. They were all of the MT27 isolates stored in the National Institute of Infectious Diseases (NIID) strain collection, which included epidemiologically related outbreak-associated isolates (Supplementary Table S1). The isolates were cultured on cyclodextrin solid medium (CSM) agar^29^ and incubated at 36 °C for 2–3 days. DNAs were extracted from the isolates by boiling and stored at − 20 °C. For whole-genome sequencing (WGS), DNAs were purified using the NucleoSpin Tissue kit (Macherey–Nagel, Germany). Novogene (Beijing, China) performed the whole-genome sequencing experiment.

### WGS

Twenty MT27 isolates collected during 2011–2018 were sequenced on the Illumina HiSeq X Ten platform with 150-bp paired-end reads performed by Novogene Co. (Supplementary Table S1). The average coverage depth of the sequencing was more than 200 × for each isolate. The sequence data were submitted to the DDBJ Sequence Read Archive (DRA) (accession no. DRA007914). All the isolates were epidemiologically unrelated cases of pertussis.

### Identification and selection of SNPs

WGS reads were mapped to the reference genome sequence of *B. pertussis* Tohama I (accession no. NC_002929.2) by the Burrows-Wheeler Aligner (BWA) software, and SNPs were detected by using the SAMtools software. A total of 269 SNPs were identified in coding sequences (including pseudogenes) between 20 sequenced isolates and the reference strain Tohama. Among the 269 SNPs, we selected a set of 20 informative SNPs (representing SNP frequencies of 7.8–49.0%) for single-base extension (SBE) assay based on 51 genome sequences of MT27 isolates: 20 sequences from this study and 31 from a previous study^17^ (Supplementary Table S2). General information on the 20 SNPs is given in Table 1.

**Table 1:** SNP markers selected for SNP genotyping using single-base extension assay.

SNP name	Position^a^	Locus ID	Gene	SNP variation	Amino acid change	Gene description	Reference
SNP2	62,982	BP0063		C/T	A100T	Hypothetical protein	^17^
SNP8	870,614	BP0841	*nuoA*	G/T	V15L	NADH-quinone oxidoreductase subunit A	^17^
SNP11	980,942	BP0944	*gyrA*	A/G	D87G	DNA gyrase subunit A	^17,30^
SNP12	1,182,054	BP1122		A/G	E47G	AsnC family transcription regulator	^17^
SNP14	1,459,272	BP1383	*flgL*	A/G	D237G	Flagellar hook-associated protein FlgL	^17^
SNP15	1,470,281	BP1394	*fliM*	C/T	Silent	Flagellar motor switch protein FliM	^17,18,28^
SNP16	1,692,984	BP1610		C/T		Pseudo	^17,25^
SNP17	1,806,441	BP1722		G/A	Silent	Hypothetical protein	^17^
SNP18	1,824,667	BP1740	*cphA*	C/T	Silent	Cyanophycin synthetase	^17^
SNP19	1,845,571	BP1758		C/T	Silent	Fatty acid desaturase	^17^
SNP20	1,890,117	BP1799	*iscS*	C/T	L131F	Cysteine desulfurase	^17,25^
SNP22	2,308,009	BP2187		G/A	Silent	Transferase	^17^
SNP24	2,395,350	BP2274		G/A	Silent	ABC transporter substrate-binding protein	^17^
SNP25	2,436,106	BP2310	*hemF*	C/T	T136I	Coproporphyrinogen III oxidase	^17^
SNP26	2,657,330	BP2509	*dapB*	A/C	T5P	4-Hydroxy-tetrahydrodipicolinate reductase	^17^
SNP28	2,884,401	BP2718		T/C	K276R	Biotin synthase	^17^
SNP30	3,326,160	BP3121		G/A	Silent	GTP-binding protein	^17^
SNP31	3,436,938	BP3224		C/T	Silent	Cytochrome oxidase	^17,18^
SNP32	3,523,618	BP3303		C/T	G56R	ADP-dependent (S)-NAD(P)H-hydrate dehydratase	^17,18^
SNP34	3,758,827	BP3547		G/A	Silent	GntR family transcriptional regulator	^17^

^a^Positions according to the plus strand on *Bordetella pertussis* Tohama genomic sequence: NC_002929.2.

Of the 20 SNP targets, 10 were coding SNPs, 9 were silent SNPs, and the remaining one was a genome SNP located in the pseudogene BP1610 (Table 1). SNP11 located in *gyrA* was associated with quinolone resistance in *B. pertussis*^30^, while SNP16 and SNP26 were previously identified as unique SNPs to epidemic isolates of Australian *B. pertussis*^25^. All 20 SNP targets were found in previous studies^17,18,25,28^.

### SNP genotyping with SBE assay

Twenty selected SNPs were divided into two groups and typed in two 10-plex PCR assays termed SNP20A and SNP20B panels. The SNP20A panel targeted SNP2, SNP11, SNP12, SNP14, SNP16, SNP18, SNP19, SNP20, SNP25, and SNP26, whereas the SNP20B targeted SNP8, SNP15, SNP17, SNP22, SNP24, SNP28, SNP30, SNP31, SNP32, and SNP34 (Table 2). Each 10-plex PCR was performed in a 15-μl reaction volume containing 7.5 μl of 2 × PCR buffer for KOD FX, 0.33 U of KOD-FX DNA polymerase (TOYOBO, Co., Ltd., Japan), 3.3 µl of 2 mM dNTPs, 1 µl of DNA sample, and 1.5 µl of primer mixture (each one at 2 µM). The primer sequences for SNP20A and SNP20B panels are shown in Table 2. PCR conditions were as follows: denaturation for 2 min at 94 °C; 35 cycles with denaturation at 98 °C for 10 s, primer annealing for 30 s at 60 °C (SNP20A) or 63 °C (SNP20B), and extension at 68 °C for 30 s; and final extension at 68 °C for 5 min. Following the PCR assay, the PCR products were treated with 5 U of shrimp alkaline phosphatase (New England Biolabs) and 2 U of exonuclease I (New England Biolabs) at 37 °C for 60 min, followed by incubation at 75 °C for 15 min for enzyme inactivation.

**Table 2:** PCR primers used for the multiple PCR panels, SNP20A and SNP20B.

Multiplex PCR panel	SNP name	PCR primer sequence (5′ → 3′)		Amplicon size (bp)
		Forward	Reverse	
SNP20A	SNP2	GTGGAGAGGCGACGCCAAAG	CTGGTGGTAACGCGTGCGAA	112
	SNP11	TATCGTCGGGGACGTCATCGG	GCCAGGCGGATTTCGGTGTAG	180
	SNP12	ATGCCAGCCTCACCAACGTC	AACCGCTCCAGCTCGCTTTC	202
	SNP14	GCCGCGATTTCCGTATCGAGTT	CGCCGTTCATGTCGATTACGGAG	150
	SNP16	GTTGGGTGCAGGAACTCAAGG	GTACGACGCGTACAGATTGTGG	143
	SNP18	CCCAATTGGCGCAATCGCCT	CGCTCCATCGTCGACCACCT	139
	SNP19	GTATCGCGAGGAGTCCGCCAA	GTCGATGGCCAGCATGGTGAG	130
	SNP20	AGGGCTTTGAAGTGACCTACCT	GGATGACGCCGATCTCGTTG	132
	SNP25	ATGCCGAATCCGACGTGTTCTG	CGGGTTTCGTTGCGGTGCTT	190
	SNP26	GGACAACCCCACCCTCGACTAC	CGAGGACGGCTTCGATCAGCA	143
SNP20B	SNP8	TGAACCTGCACCCGTATTTCCCC	TCGAACTTCATCCGCGCGTCT	172
	SNP15	GCAGCACGTTGCGCATGTGG	CGGCGAAAGCGACGAGAAGCAG	152
	SNP17	CGAGGTGTCCGACCTGGAAGTG	GGAAACCCTGGTCGTCGCGAAA	134
	SNP22	GCCTTTCCGGATCCAGGCTTCA	GAAGGGGCATTCGCAGGCCA	130
	SNP24	GGATCGCCAGCACGGCGAAG	GAAGCCGTGTTCCCGTCCGAAG	160
	SNP28	CACAGCGCCTGCTCGCTGTC	TGCCCATCAACAACCTGGTGCAA	172
	SNP30	CTTGCCTTCGGCGCGCATCT	GCCGGTGTCATCCACACCGACT	129
	SNP31	CATCATGCGGCCGCGCTCTT	GCCCCGACGATCCCTCGTTCT	149
	SNP32	TCGATGCCCAGGCCCGCTT	AGCACCTGCCCGCCCTGTT	256
	SNP34	GAACGTGGGCGACAAATTGCCT	CCGACGTTCTGCACGTCGCT	192

Each 10-plex SBE assay was carried out in a 10-µl reaction volume containing 2.5 µl of SNaPshot Multiplex Ready Reaction Mix (Applied Biosystems), 2 µl of 5 × sequencing buffer (Applied Biosystems), 0.7 µl of cleaned PCR products, and optimized concentrations of ten SBE primers. The sequences and concentrations of SBE primers for SNP20A and SNP20B panels are listed in Table 3. Each SBE reaction was performed for 25 cycles of denaturation at 94 °C for 10 s, primer annealing at 50 °C for 5 s, and extension at 60 °C for 30 s. Unincorporated fluorescently labeled ddNTPs were removed by addition of 1 U of shrimp alkaline phosphatase and incubation at 37 °C for 60 min, followed by incubation at 75 °C for 15 min to inactivate the enzyme. The SBE products (0.5 µl) were mixed with 9 µl of Hi-Di formamide and 0.5 µl of GeneScan 120 LIZ size standard (Applied Biosystems). After heat denaturation for 5 min at 95 °C and rapid cooling on ice, the SBE products were separated using the Applied Biosystems 3130xl Genetic Analyzer with dye set E5. The data analysis was performed with GeneMapper software (Applied Biosystems), and the resulting data were concatenated to produce a 20-position SNP profile for each isolate tested.

**Table 3:** Single-base extension (SBE) primers used for the multiplex PCR panels, SNP20A and SNP20B.

Multiplex PCR panel	SNP name	SBE primer sequence (5′ → 3′)^a^	Orientation^b^	Primer size (nt)	Primer conc (µM)
SNP20A	SNP2	ATTTCAGATCTTGTCGCAGCG	R	21	0.05
	SNP11	ctgactACGGCGACCAGTCGGTATACG	F	27	0.05
	SNP12	actgactgactgactCCCGCGTCAAGGCCCTGG	F	33	0.2
	SNP14	gactgactgactgactgactACGCGCGATGCGGTGACCG	F	39	0.2
	SNP16	actgactgactgactgactgactgactCGCGCCCAGGTCGGTACG	R	45	0.6
	SNP18	gactgactgactgactgactgactgactgactTCTGCACCAGGCGCGCCAC	F	51	0.2
	SNP19	actgactgactgactgactgactgactgactgactGGCTCGAACGCAACATCTATTC	F	57	0.2
	SNP20	actgactgactgactgactgactgactgactgactgactgactATGTCCAGGACGATGGTCTG	F	63	0.8
	SNP25	actgactgactgactgactgactgactgactgactgactgactgactgactGCGGCGGGCTCGACCTCA	F	69	0.2
	SNP26	ctgactgactgactgactgactgactgactgactgactgactgactgactgactCTCAAGTAAATGACGCAAGCC	F	75	0.3
SNP20B	SNP8	ctgactgactgactgactgactgactgactgactgactgactgactgactgactgactgactCCCGTCCTGCTGTTTATCCTG	F	83	0.3
	SNP15	ctgactgactAGCTGAGGTCGTAGGCGCG	F	29	0.5
	SNP17	ctgactgactgactGTGCAGGGGCAGGAAGTCGTG	R	35	0.4
	SNP22	actgactgactgactgactGCCATTCGAGGACTACGACAGC	R	41	0.1
	SNP24	actgactgactgactgactgactgactGTCAAGGGCACCAAGCATCT	R	47	0.1
	SNP28	ctgactgactgactgactgactgactgactgactATCACCATGCCGCTGGCCA	R	53	0.2
	SNP30	tgactgactgactgactgactgactgactgactgactCGATGAAGTCTTCGTAGGCGAT	F	59	0.1
	SNP31	tgactgactgactgactgactgactgactgactgactgactgactAACAGCCCGACGAAAACCAG	F	65	0.2
	SNP32	tgactgactgactgactgactgactgactgactgactgactgactgactTTGCCGGCCCCCACTTTCAGTC	F	71	0.4
	SNP34	gactgactgactgactgactgactgactgactgactgactgactgactgactgactCTGCGAGAAATTCGGCGTGTC	F	77	0.6

^a^The sequences in uppercase and lowercase represent the target specific sequence and neutral sequence, respectively.

^b^Orientation of primer according to the genomic sequence of *Bordetella pertussis* Tohama: NC_002929.2. *F* forward; *R* reverse.

### Analysis of allele sequence for virulence-associated genes

Four virulence-associated allelic genes, *ptxP*, *ptxA*, *prn,* and *fim3*, were analyzed using PCR-based sequencing, as previously described^4^. The allele profiles were expressed as *ptxP*/*ptxA*/*prn*/*fim3* based on the combination of the allelic genes.

### MLVA genotyping

PCR-based MLVA targeting VNTR1, VNTR3a, VNTR3b, VNTR4, VNTR5, and VNTR6 loci was performed as previously described^4,31,32^. The MT27 strain was defined as carrying eight tandem repeats in VNTR1, seven in VNTR3a, zero in VNTR3b, seven in VNTR4, six in VNTR5 and seven in VNTR6 as described previously^14^.

### Data analysis

Analysis of *B. pertussis* genome data was performed with CLC Genomics Workbench version 8.5.4 (CLC Bio, Denmark) using the reference genome sequence of *B. pertussis* Tohama I (accession no. NC_002929.2). CSI Phylogeny 1.4 was used to call SNPs from WGS data and infer phylogeny based on the concatenated alignment of SNPs^33^. Maximum parsimony tree was constructed using MEGA7 version 7.0.26^34^. Simpson’s diversity index (DI) was calculated as described by Hunter and Gaston^35^ using the online tool available at http://www.comparingpartitions.info/.

## Results

### Characteristics of MT27 isolates

Among the 237 *B. pertussis* MT27 isolates tested, 208 (88%) carried the allele profile of *ptxP3/ptxA1*/*prn2*/*fim3A*, and were the most predominant subtype. The remaining 29 isolates carried the minor allele profiles, *ptxP3*/*ptxA1*/*prn2*/*fim3B* (n = 19, 8%), *ptxP3*/*ptxA1*/*prn9*/*fim3A* (n = 5, 2%), *ptxP3*/*ptxA1*/*prn9*/*fim3B* (n = 2, 0.8%) *ptxP3*/*ptxA1*/*prn3*/*fim3A* (n = 1, 0.4%), *ptxP3*/*ptxA1*/*prn14*/*fim3A* (n = 1, 0.4%), and *ptxP1*/*ptxA1*/*prn15*/*fim3A* (n = 1, 0.4%). The *B. pertussis* MT27 isolate carrying *ptxP1* allele was identified for the first time in Japan.

### Discriminatory power and reliability of the SNP genotyping

**Figure 1:**
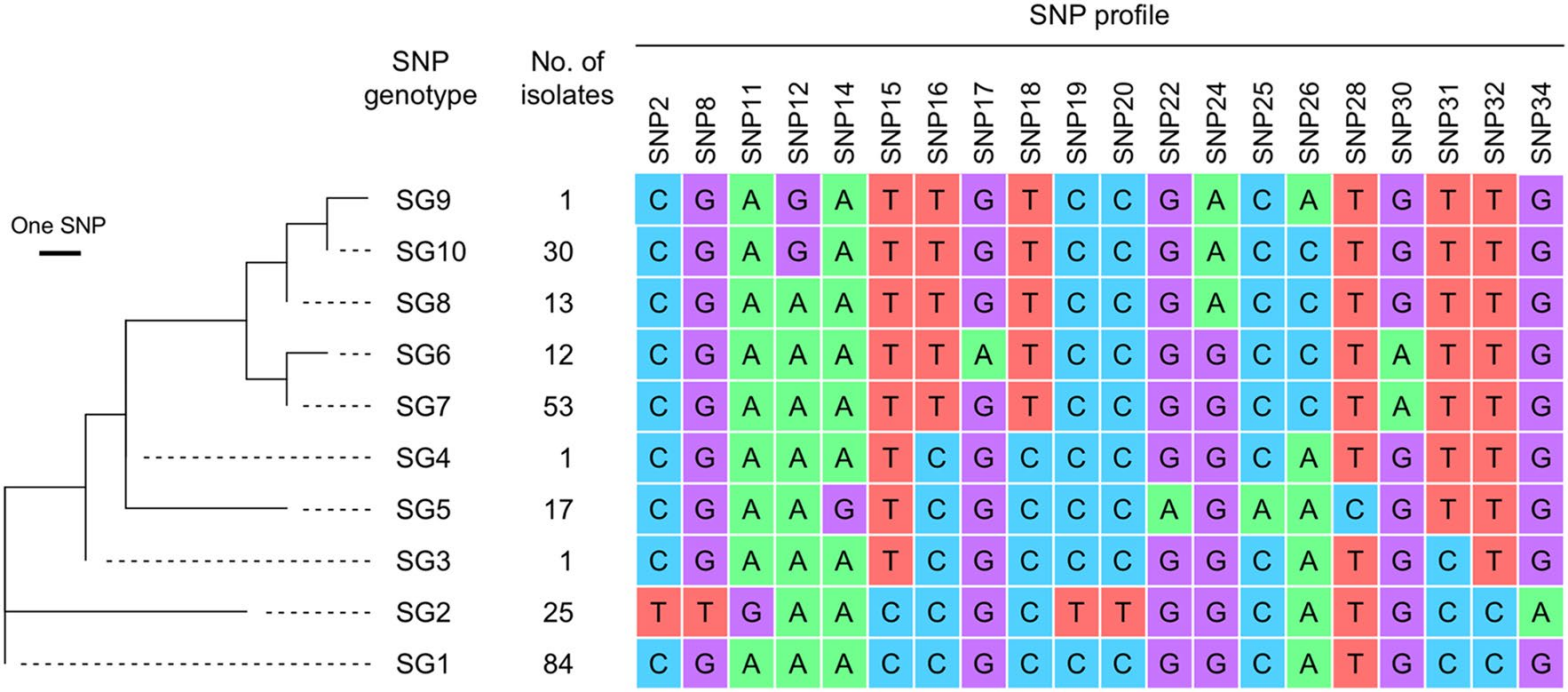
Maximum parsimony tree of *Bordetella pertussis* MT27 isolates based on 20-position SNP profile. A total of 237 isolates collected in Japan were analyzed by SNP genotyping and were divided into 10 SNP genotypes (SGs). The phylogenetic tree was constructed using MEGA7. The branch length is proportional to the number of SNP differences. The 20-position SNP profiles are shown on the right.

A total of 237 MT27 isolates were divided into 10 SNP genotypes (SGs) with a Simpson’s DI of 0.79 (95% CI 0.76–0.82) (Fig. 1). Four SGs, SG1, SG2, SG7, and SG10 were the prevalent genotypes, representing 35%, 11%, 22% and 13% of the isolates, respectively. Other SGs, SG3–SG6, SG8, and SG9 were minor genotypes (representing 0.4–7.2% of the isolates). SG2 had a unique 20-position SNP profile because six SNPs (SNP2, SNP8, SNP11, SNP19, SNP20, and SNP34) were specifically associated with SG2. In contrast, five SGs (SG6–SG10) formed a phylogenetic cluster on the maximum parsimony tree (Fig. 1).

In reference to the 51 sequenced isolates, all 20-position SNP profiles identified by the SNP genotyping were identical to those obtained from WGS data. The SBE-based SNP genotyping showed high reliability.

### Comparison with whole-genome SNP genotyping

**Figure 2:**
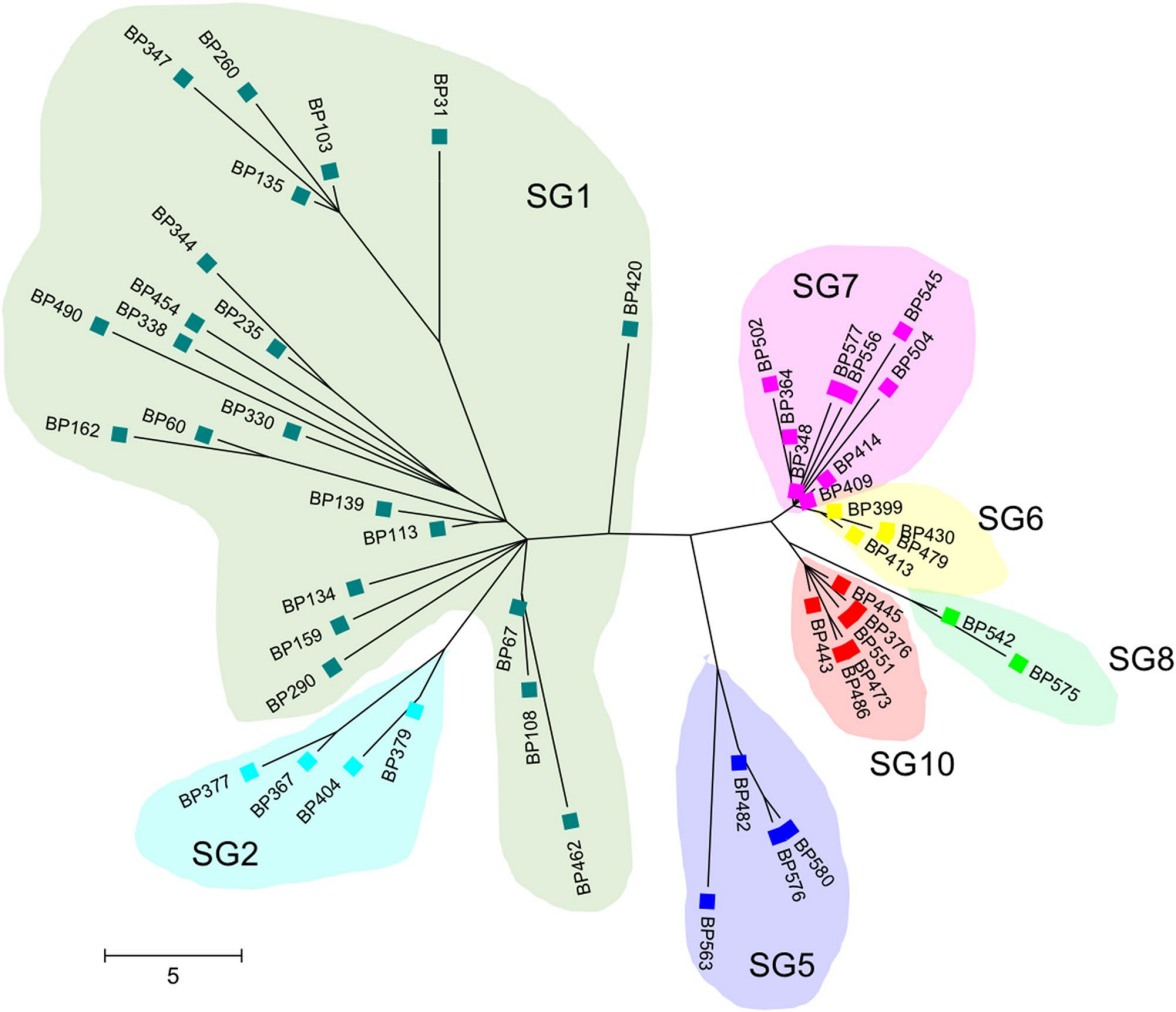
Maximum parsimony tree of 51 *Bordetella pertussis* MT27 isolates based on whole-genome SNPs. A total of 254 SNPs were identified among 51 genome-sequenced MT27 isolates using CSI Phylogeny 1.4. The radial tree was constructed using MEGA7. SBE-based SNP genotypes are highlighted by different colors. Scale bar indicates SNPs.

Figure 2 shows the phylogenetic tree of the 51 MT27 isolates based on whole-genome SNPs. The tree was constructed based on 254 SNPs that were identified among the genome-sequenced isolates using CSI Phylogeny 1.4 (Supplementary Table S3), and it was compared with SBE-based SNP genotyping with 20 SNP targets. Almost all isolates (49/51) were distinguished by the whole-genome SNP genotyping. Twenty-two SG1 isolates collected mainly in the 2000s were not grouped into a small cluster, but other isolates (SG2, SG5–SG8, and SG10, which were mostly collected in the 2010s) were grouped into each category of the SBE-based SNP genotypes. The SG1 isolates had a mean of 20.3 SNP differences between them (intra-clade SNP distance, 2–36 SNPs), whereas other isolates had small SNP differences (SG2, 8.2 differences; SG5, 7.8; SG6, 1.8; SG7, 5.9; SG8, 5.0; SG10, 3.8). Whole-genome SNP genotyping illustrated that the old SG1 strain had high SNP diversity. Except for SG1 isolates, there was good agreement between the SBE-SNP genotyping and the whole-genome SNP genotyping. The reference strain Tohama I (MLVA type, MT83) belonged to SG1.

### Temporal changes in SNP genotypes and genotypic diversity

**Figure 3:**
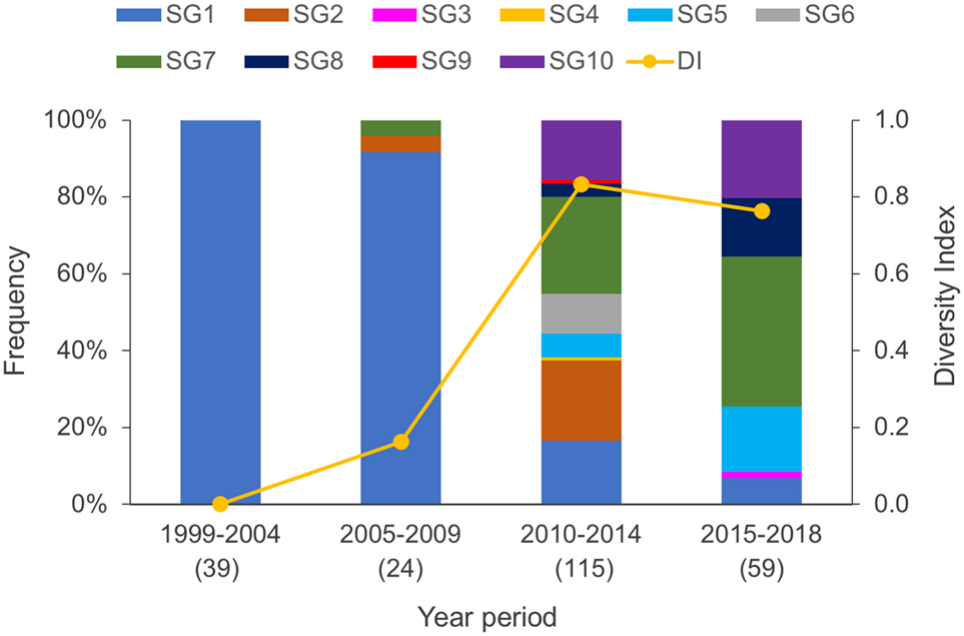
Frequency of SNP genotypes and genotypic diversity of *Bordetella pertussis* MT27 isolates collected in Japan during 1999–2018. Simpson’s diversity index (DI) was examined with the frequencies of SNP genotypes (SGs) within four time periods. Numbers in parentheses indicate the number of isolates analyzed.

Figure 3 shows the temporal trend of the frequency of SGs during four time periods. The MT27 isolates from 1999 to 2004 (n = 39) belonged to only SG1, whereas those from 2005 to 2009 (n = 24) belonged to SG1, SG2, and SG7. Further, the isolates from 2010 to 2014 (n = 115) belonged to nine SGs (SG1, SG2, and SG4–SG10), and those from 2015 to 2018 (n = 59) belonged to six SGs (SG1, SG3, SG5, SG7, SG8, and SG10). The frequency of the SG1 strain markedly decreased in the 2010s, while the frequency of the SG7 strain significantly increased. Simpson’s DI for the SGs was zero in 1999–2004, 0.16 in 2005–2009, 0.83 in 2010–2014, and 0.76 in 2015–2018. The genotypic diversity of MT27 isolates increased markedly in the 2010s.

### Relationship between SNP genotypes and virulence-associated allelic genes

Seven allele profiles were identified in the MT27 isolates tested. The isolate carrying *ptxP3*/*ptxA1*/*prn2*/*fim3A* was found to be predominant in all SGs (Table 4). The isolates carrying other allele profiles were in SG1, SG7, and SG10, but the numbers of isolates were much smaller compared with isolates carrying the predominant allele profile. There were no SGs predominantly related to the minor allele profiles; however, all isolates carrying *fim3B* were in SG1.

**Table 4:** Relationship between SNP genotypes and virulence-associated allelic gene profiles in 237 *Bordetella pertussis* MT27 isolates.

SNP genotype	No. of isolates	No. of isolates with the allele profile						
		*ptxP3*/*ptxA1*/*prn2*/*fim3A*	*ptxP3*/*ptxA1*/*prn2*/*fim3B*	*ptxP3*/*ptxA1*/*prn9*/*fim3A*	*ptxP3*/*ptxA1*/*prn9*/*fim3B*	*ptxP3*/*ptxA1*/*prn3*/*fim3A*	*ptxP3*/*ptxA1*/*prn14*/*fim3A*	*ptxP1*/*ptxA1*/*prn15*/*fim3A*
SG1	84	61	19	1	2			1
SG2	25	25						
SG3	1	1						
SG4	1	1						
SG5	17	17						
SG6	12	12						
SG7	53	51				1	1	
SG8	13	13						
SG9	1	1						
SG10	30	26		4				

### Application of SNP genotyping to outbreak-associated isolates

MT27 isolates from pertussis outbreaks were genotyped (Table 5). Fifteen outbreak-associated isolates were collected in Miyazaki prefecture^36^, and all exhibited the same genotype of SG2. Similarly, all isolates from Toyama and Niigata prefectures (n = 4 each) belonged to the same SG10. Of the 12 isolates from Nagano prefecture, 1 and 11 were SG5 and SG7, respectively. The SG5 strain was not closely related to the SG7 strain since the SG5 strain had eight different SNPs in the 20-position SNP profile as compared with the SG7 strain. Together, all except one isolate were classified into the same SNP genotypes for each outbreak.

**Table 5:** SNP genotypes of *Bordetella pertussis* MT27 isolates collected from pertussis outbreaks.

Location (Prefecture)	Year	Allelic gene profile of isolates	No. of isolates with the SNP genotype			
			SG2	SG5	SG7	SG10
Miyazaki	2010–2011	*ptxP3*/*ptxA1*/*prn2*/*fim3A*	15			
Toyama	2015	*ptxP3*/*ptxA1*/*prn2*/*fim3A*				4
Nagano	2016	*ptxP3*/*ptxA1*/*prn2*/*fim3A*		1	11	
Niigata	2018	*ptxP3*/*ptxA1*/*prn9*/*fim3A*				4

### SNP genotyping for non-MT27 isolates

A total of 104 non-MT27 isolates were classified into 4 SGs (SG1, SG3, SG7, and SG10) with a Simpson’s DI of 0.21 (95% CI 0.11–0.31) (Supplementary Table S4). Ninety-two isolates (89%) belonged to SG1 and the remainder (11%) to SG3, SG7, and SG10. The isolates belonging to SG3, SG7, and SG10 were recent isolates collected in the 2010s. Overall, the genotypic diversity was much lower than that of MT27 isolates.

### SNP genotyping for Taiwanese isolates

We genotyped *B. pertussis* isolates collected in Taiwan during 2010–2019. Of 48 isolates, 30 (62.5%) were MT27 and 18 (37.5%) were non-MT27 (Supplementary Table S5). The MT27 isolates were subdivided into 5 SGs (SG1, SG3, SG5, SG7, and SG11) with a Simpson’s DI of 0.69 (95% CI 0.56–0.82). Fifteen MT27 isolates belonged to SG1 and the remainder to SG3 (n = 4), SG5 (n = 1), SG7 (n = 3), and SG11 (n = 7). Similarly, the non-MT27 isolates were classified into 4 SGs (SG1, SG3, SG7, and SG11) with a Simpson’s DI of 0.73 (95% CI 0.61–0.85). The SG11 was not found in the Japanese isolates tested including non-MT27 isolates, and its 20-position SNP profile was CGAAATTGTCCGGCCTGTTG.

### SNP genotypes of US isolates

We determined 20-position SNP profiles of MT27 isolates collected in the US during 2000–2013, based on their complete genome sequences available in the GenBank database^16^. One hundred twenty-two MT27 isolates were classified into the 6 SGs, i.e., SG1 (n = 89), SG3 (n = 5), SG4 (n = 6), SG7 (n = 20), SG11 (n = 1), and SG12 (n = 1). The minor SG12 was novel and its 20-position SNP profiles was CGAAATCGCCCAGCATGTTG. Simpson’s DI for the SGs was zero in 2000–2004, zero in 2005–2009, and 0.64 in 2010–2013 (Supplementary Fig. S1). The genotypic diversity of the US isolates markedly increased in the 2010s, similar to that of Japanese MT27 isolates.

## Discussion

We developed an SBE-based SNP genotyping for the *B. pertussis* epidemic strain MT27 and evaluated its applicability. The data presented here show that Japanese MT27 isolates were subdivided into ten SNP genotypes and that the genotypic diversity of MT27 isolates markedly increased in the 2010s. Moreover, almost all outbreak-associated MT27 isolates were classified into the same SNP genotypes for each outbreak. The SNP genotyping method allows for subtyping of the recently circulating MT27 strain in routine surveillance and outbreak investigations. This is supported by analyses of Taiwanese and US isolates.

In this study, we demonstrated that the genotypic diversity with 20 SNP targets rapidly increased among Japanese MT27 isolates in the 2010s. One possible cause for the increased diversity is the pertussis epidemic. In Japan, a nationwide epidemic of pertussis occurred between 2008 and 2010, and the frequency of the MT27 strain increased during the epidemic period^4^. In this study, the SG1-MT27 strain was predominant in 2005–2010, and the genotypic diversity of the MT27 strain increased markedly in the 2010s (Fig. 3). These observations indicate that the SG1–MT27 strain was rapidly replaced with other genotypes (non-SG1) during and after the epidemic. We therefore speculate that *B. pertussis* has evolved by increasing diversity during pertussis epidemics. A previous study showed that the *B. pertussis* population (genotype prevalence) increased with changes in vaccine usage (coverage and schedule)^17^. In Japan, acellular pertussis vaccines (ACVs) were introduced in 1981 instead of whole-cell pertussis vaccines, and there were no changes in the vaccine usage after 1996. Therefore, ACVs were not directly associated with the increased genotypic diversity.

Interestingly, the rapid increase in SNP diversity was also observed among the US MT27 isolates in the early 2010s (Supplementary Fig. S1). In Japan and the US, the frequency of the SG1 strain decreased with time, whereas that of the SG7 strain markedly increased. The Japanese SG7 isolates (n = 53) were collected from 7 out of 8 districts, indicating that the emergence of the SG7 strain occurred nationwide. Similarly, the US SG7 isolates (n = 20) were collected from 13 states^16^. These observations suggest that the SBE-based SNP genotyping may also apply to recently circulating MT27 strain collected in countries other than Japan. In fact, we confirmed here that the SBE-based SNP genotyping was applicable to recent Taiwanese MT27 isolates. For the Japanese and US SG7 strains, our preliminary analysis with whole-genome SNPs shows that most US SG7 isolates are slightly different from Japanese SG7 isolates (inter-clade SNP distance, 4 SNPs), but one isolate was found to be 100% identical to Japanese SG7 isolates. Further genetic studies are needed to characterize the increasing non-SG1 strains, especially SG7.

The *B. pertussis* population has significantly changed worldwide in the last 60 years^18^. Strains carrying the virulence-associated allelic genes *ptxP3* and *fim3A* have emerged and expanded, and those carrying *ptxP1* and *fim3A* had decreased. More recently, *ptxP3* strains carrying *fim3B* (alias *fim3-2*) have expanded. In this study, most Japanese MT27 isolates carried *ptxP3* and *fim3A* and were classified into 10 SGs based on the 20-position SNP profile (Supplementary Fig. S2). In contrast, all MT27 isolates carrying *fim3B* (n = 21) were interestingly grouped into only SG1, implying that the *fim3B* strains are not included in non-SG1 groups. The *fim3B* strain has increased globally and has the potential to cause recent pertussis epidemics^4,23^. Our SBE-based SNP genotyping could be helpful to identify the emerging *fim3B* strain.

Here, whole-genome SNP genotyping showed that non-SG1 isolates clustered into each group of the SBE-based SNP genotypes, but SG1 isolates did not (Fig. 2). Most SG1 isolates were collected in the 2000s, whereas non-SG1 isolates were in the 2010s (Fig. 3). This suggests that the new non-SG1 strains have clonally expanded, but their genetic diversity may further increase with time, similar to the old SG1 strain. Thus, continuous genome surveillance is required for accurate subtyping of *B. pertussis* MT27 strain, especially for non-SG1 strains.

In the present study, we applied the SBE-based SNP genotyping to outbreak-associated MT27 isolates. Among 12 isolates from the Nagano outbreak, one isolate had a genotype (SG5) different from those of the other 11 isolates (SG7) (Table 5). In the 20-position SNP profile, 8 SNPs were different between the SG5 and SG7 isolates. This indicates that the SG5 isolate was collected from a sporadic case that was not associated with the outbreak. In outbreak investigations, strain typing contributes to understanding bacterial transmission route(s), and rapid typing is important to take countermeasures to prevent further spread of the bacteria. Our SBE-based SNP genotyping is amenable to high-throughput analysis using 96-well plates. Starting from DNA extracts, the SBE-based SNP genotyping was able to analyze 96 isolates within two days, contributing to rapid genotyping, which would be critical in outbreak investigations involving large numbers of isolates.

Culture of *B. pertussis* has limited sensitivity for previously vaccinated persons, older children, adolescents, and adults^37,38^. Therefore, molecular strain typing (MLVA and/or MLST) is performed not only on the bacterial isolates, but also on DNA extracts from clinical specimens such as nasopharyngeal swabs^22,39–41^. Our SBE-based SNP genotyping includes an initial PCR step that amplifies the region around each SNP target. We, therefore, tested the applicability of the SNP genotyping to clinical specimens. Of the 20 clinical specimens that were positive for *B. pertussis* by a nucleic acid amplification test, 13 (65%) were genotyped by the direct SNP genotyping (Supplementary Table S6). The remaining seven clinical specimens had lower bacterial loads than those of the genotyped specimens (mean Ct values, 23.7 versus 20.4; *P* < 0.01, Mann Whitney *U* test), indicating that improvements in the initial PCR conditions (including primer design) are required to increase the analytical power. Although there is room for improvement, SBE-based SNP genotyping has the potential to be directly applicable to clinical specimens.

In this study, we also tested the applicability of the SBE-based SNP genotyping to *B. pertussis* non-MT27 strains. Our data demonstrated that most Japanese non-MT27 isolates (89%) belonged to SG1, showing low genotypic diversity (Simpson’s DI, 0.21) (Supplementary Table S4). In contrast, high genotypic diversity was seen for Taiwanese non-MT27 isolates (Simpson’s DI, 0.73), although the number of isolates tested was small (Supplementary Table S5). Further analyses are needed for the applicability of the SBE-based SNP genotyping to non-MT27 isolates. A limitation of our SNP genotyping targeting 20 SNPs is that its discriminatory power is lower than that of whole-genome SNP genotyping (targeting over 250 SNPs). Therefore, our simple SNP genotyping cannot provide true genetic relationships among MT27 isolates, especially for SG1 strain of MT27; whole-genome SNP genotyping is required for this purpose.

In conclusion, the present study describes the successful development of a simple and rapid SNP genotyping for the subtyping of *B. pertussis* MT27 isolates. Since the MT27 strain is the predominant strain in most industrialized countries, our SNP genotyping can serve as a novel alternative to whole-genome SNP genotyping in routine surveillance and outbreak investigations.

## Supplementary Information

Supplementary Information 1.

Supplementary Information 2.

## Data Availability

All data generated or analysed during this study are included in this published article and its Supplementary Information files.
