# Supplementary material for: Rapid and simple SNP genotyping for Bordetella pertussis epidemic strain MT27 based on a multiplexed single-base extension assay
Source: Sci Rep. 2021 Mar 1;11:4823. doi: 10.1038/s41598-021-84409-0 (PMC7921669; doi:10.1038/s41598-021-84409-0)
Supplement: Supplementary file 2 — Supplementary Information 2. [file 41598_2021_84409_MOESM2_ESM.docx]

**Supplementary Information**

**Rapid and simple SNP genotyping for *Bordetella pertussis* epidemic strain MT27 based on a multiplexed single-base extension assay**

Kazunari Kamachi^1^*, Shu-Man Yao^2^, Chuen-Sheue Chiang^2^, Kentaro Koide^1^, Nao Otsuka^1^ & Keigo Shibayama^1^

^1^ Department of Bacteriology II, National Institute of Infectious Diseases, Tokyo, Japan

^2^ Center for Diagnostics and Vaccine Development, Centers for Disease Control, Taipei, Taiwan


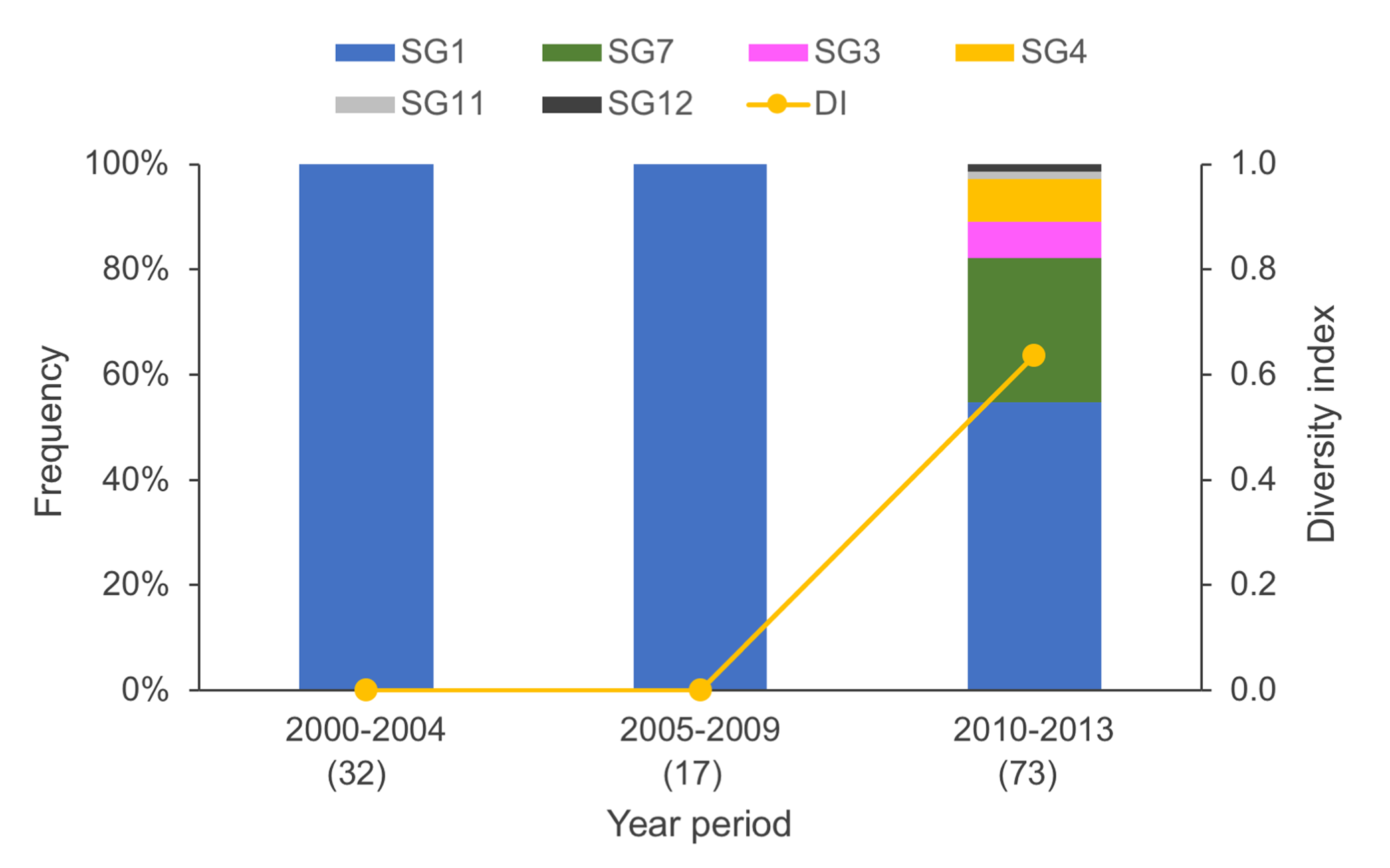


**Supplementary Figure S1.** Frequency of SNP genotypes and genotypic diversity of *Bordetella pertussis* MT27 isolates collected in the US during 2000−2013. Twenty-position SNP profiles of the MT27 isolates were determined based on their complete genome sequences available in the GenBank database. Simpson’s diversity index (DI) was examined with the frequencies of SNP genotypes (SGs) within three time periods. Numbers in parentheses indicate the number of isolates analyzed.


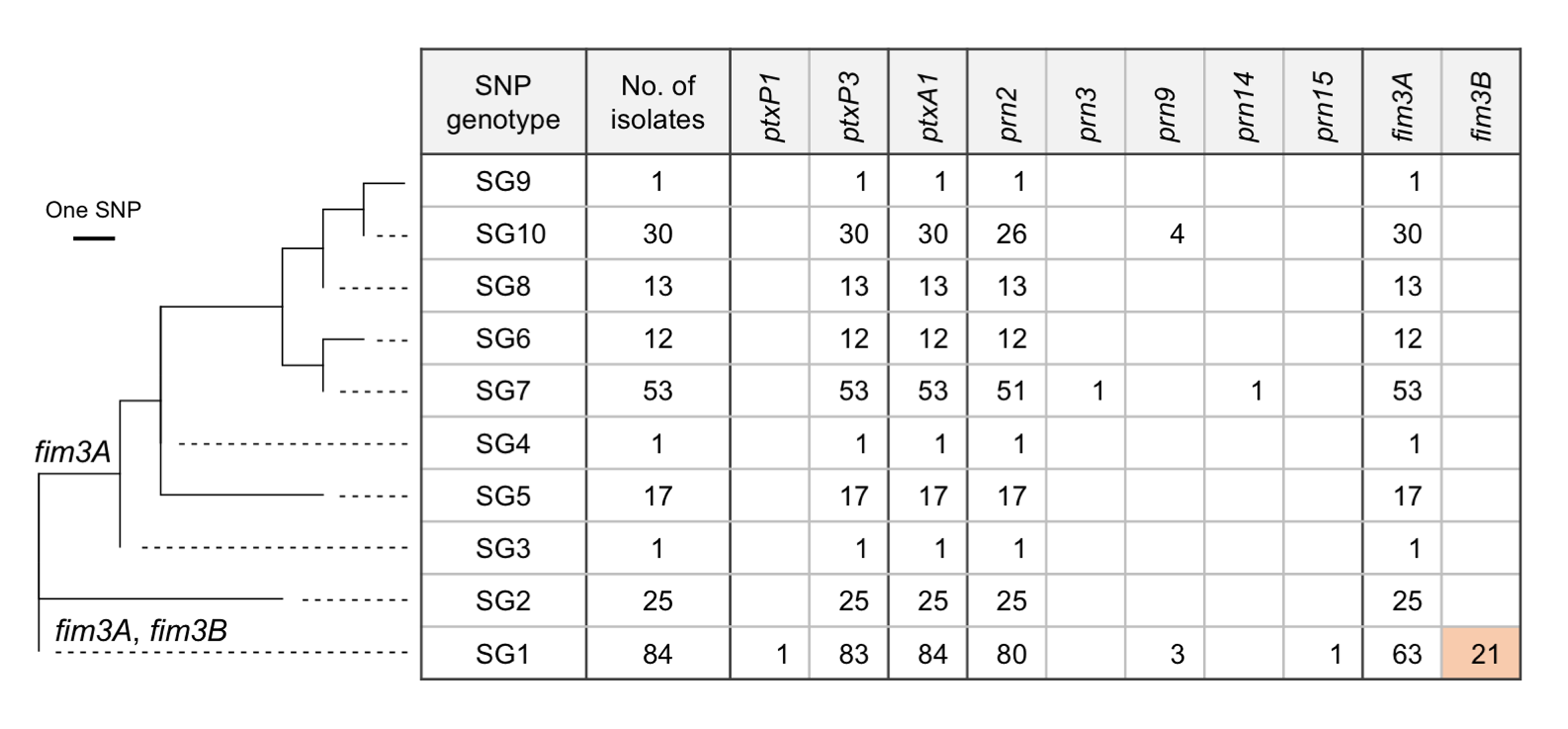


**Supplementary Figure S2.** Relationship between single nucleotide polymorphism (SNP) genotypes and virulence-associated allelic genes (*ptxP*, *ptxA*, *prn*, and *fim3*) in 237 *Bordetella pertussis* MT27 isolates. The numbers refer to the number of isolates for each SNP genotype (SG). The maximum parsimony tree based on 20-position SNP profile is shown on the left. The phylogenetic tree was constructed using MEGA7.
